# Endothelial lipase variant T111I does not alter inhibition by angiopoietin-like proteins

**DOI:** 10.1038/s41598-024-54705-6

**Published:** 2024-02-20

**Authors:** Kelli L. Sylvers-Davie, Kaleb C. Bierstedt, Michael J. Schnieders, Brandon S. J. Davies

**Affiliations:** 1 Department of Biochemistry and Molecular Biology, University of Iowa, 169 Newton Rd., PBDB 3326, Iowa, IA 52242 USA; 2 Fraternal Order of Eagles Diabetes Research Center, University of Iowa, Iowa, IA 52242 USA; 3 Department of Biomedical Engineering, University of Iowa, Iowa, IA 52242 USA

**Keywords:** Biochemistry, Molecular biology, Molecular medicine

## Abstract

High levels of HDL-C are correlated with a decreased risk of cardiovascular disease. HDL-C levels are modulated in part by the secreted phospholipase, endothelial lipase (EL), which hydrolyzes the phospholipids of HDL and decreases circulating HDL-C concentrations. A 584C/T polymorphism in *LIPG*, the gene which encodes EL, was first identified in individuals with increased HDL levels. This polymorphism results in a T111I point mutation the EL protein. The association between this variant, HDL levels, and the risk of coronary artery disease (CAD) in humans has been extensively studied, but the findings have been inconsistent. In this study, we took a biochemical approach, investigating how the T111I variant affected EL activity, structure, and stability. Moreover, we tested whether the T111I variant altered the inhibition of phospholipase activity by angiopoietin-like 3 (ANGPTL3) and angiopoietin-like 4 (ANGPTL4), two known EL inhibitors. We found that neither the stability nor enzymatic activity of EL was altered by the T111I variant. Moreover, we found no difference between wild-type and T111I EL in their ability to be inhibited by ANGPTL proteins. These data suggest that any effect this variant may have on HDL-C levels or cardiovascular disease are not mediated through alterations in these functions.

## Introduction

High-density lipoproteins play an important role in cholesterol homeostasis by participating in reverse cholesterol transport, a process in which excess cholesterol is loaded onto HDL to be removed from peripheral tissues and macrophages then transported back to the liver for excretion^1^. Reverse cholesterol transport, in combination with additional anti-atherogenic properties of HDL, including anti-inflammatory, anti-apoptotic, and anti-thrombotic properties, are likely responsible for the correlation between HDL levels and cardiovascular health^2–5^. Endothelial lipase (EL), a secreted lipase derived from endothelial cells in highly vascularized tissues, hydrolyzes the phospholipids that make up the outer shell of HDL at the sn-1 position^6^. This remodeling of the HDL membrane accelerates clearance of HDL by downstream receptors, decreasing plasma HDL-cholesterol (HDL-C) levels^7^. The link between EL and HDL levels has been demonstrated and validated in mice, where EL-deficiency results in increased HDL-C and HDL phospholipids, and overexpression of EL results in decreased HDL-C^8–10^.

Several polymorphisms in *LIPG,* the gene that encodes endothelial lipase, have been identified in human patients. A common polymorphism located in exon 3, 584C/T (rs2000813), results in an amino acid substitution, Thr111Ile^11^. The T111 residue is conserved in humans, primates, and rats, but not in mice or rabbits. Although this polymorphism was first identified in a 2002 study of individuals with elevated HDL-C levels^11^, subsequent studies have produced mixed results when attempting to link this variant to altered HDL concentrations. Several studies have found an association between Thr111Ile and increased HDL levels and reduced risk of coronary heart disease risk^10,12–17^, but other studies failed to find any correlation^18–23^. In biochemical studies, EL T111I appears to have similar phospholipase activity as wild-type EL^23,24^. Thus, if and how this mutation affects HDL levels and other cardiac risk factors remains unclear.

The major endogenous regulator of EL activity is the hepatokine, angiopoietin-like 3 (ANGPTL3). By itself, ANGPTL3 is a potent inhibitor of EL^25^, and when in complex with ANGPTL8, it can also potently inhibit lipoprotein lipase (LPL), a lipase closely related to EL^26–31^. ANGPTL3 inhibition of EL results in increased levels of HDL and HDL-C^25,32^. An altered ability of EL T111I to be inhibited by ANGPTL3 could explain the differences in HDL-C levels sometimes observed with this allele. However, the ability of ANGPTL3 to inhibit the activity of the T111I variant of EL has not yet been examined.

In this study, we evaluated the EL variant with a T111I substitution for phospholipase activity, stability, and ability to be inhibited by ANGPTL proteins.

## Results

### Phospholipase activity of EL T111I

There are conflicting reports of the association of the EL T111I variant with HDL levels^10–20,22,23^. We reasoned that if the T111I mutation does alter HDL-C levels, these alterations could be mediated by decreases in EL activity or protein stability, or an increase in susceptibility to ANGPTL3 inhibition.

**Figure 1:**
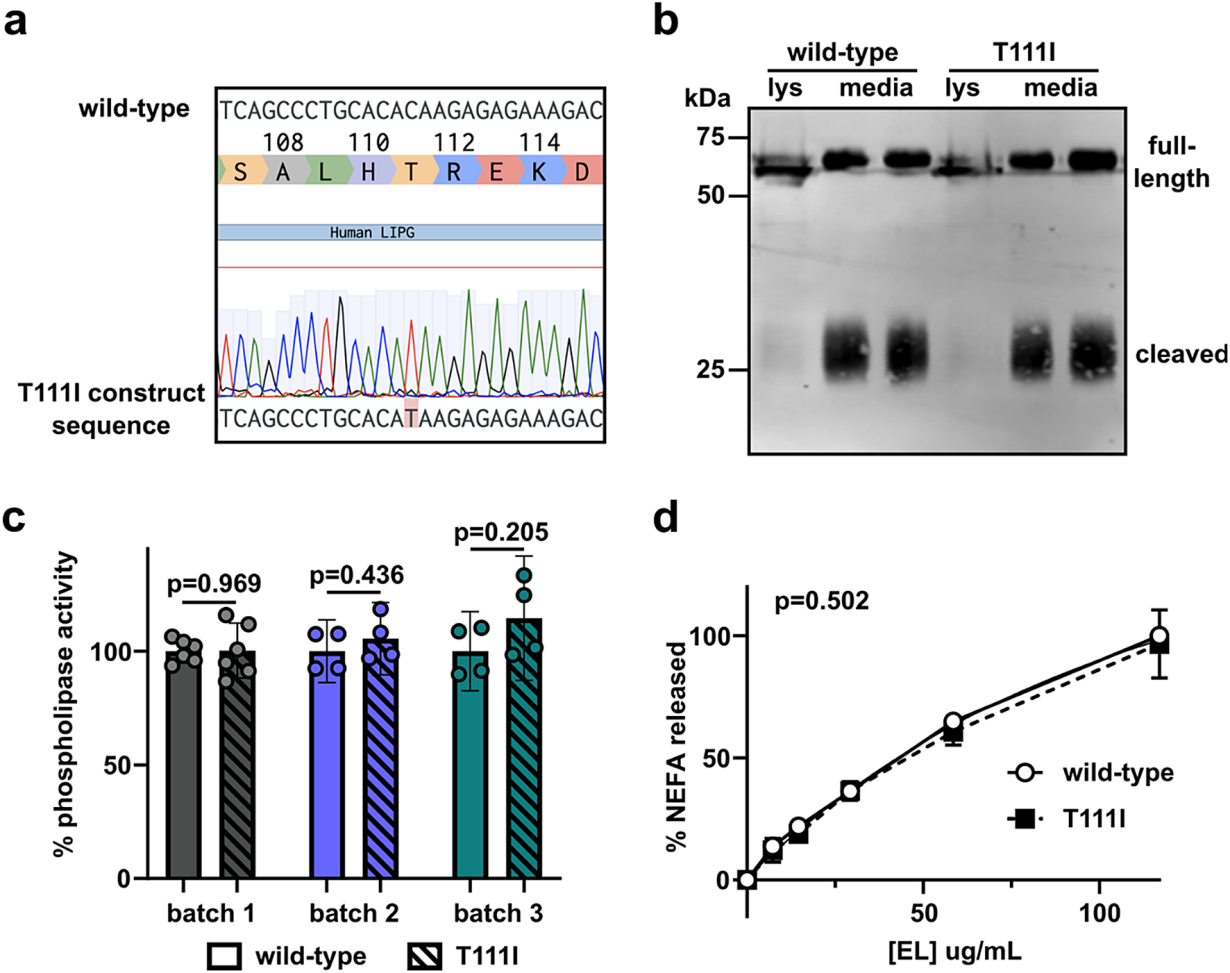
Expression and activity of the EL T111I mutation. (**a**) Sequencing data showing successful generation of the 584C/T mutation in human LIPG. (**b**) Expression and secretion of wild-type and T111I EL. Western blot shows expression of EL in the lysate (lys) and media of 293 T cells transfected with wild-type or T111I EL. Uncropped blot shown in Supplemental Fig. 1a. (**c**) Activity of three independently collected batches of WT and T111I EL. For each batch constructs expressing WT and T111I EL were transfected at the same time. After matching protein levels via western blot intensity, phospholipase activity of the conditioned media was measured using a fluorescence-based phospholipase activity assay. Bars show mean ± SD. P values determined using student t test. (**d**) Phospholipase activity of EL as measured by the release of NEFAs from HDL. The indicated concentrations of EL were combined with human HDL for 60 min at 37 °C. Points represents 3 independent experiments (mean ± SD). P value determined by one phase decay least squares fit.

To address the potential effect of the T111I mutation on EL function, we generated expression constructs coding for wild-type and T111I mutant EL **(**Fig. 1a). When these constructs were transiently expressed in HEK 293 T cells, we found that expression, secretion, and cleavage of the EL T111I mutant was similar to wild-type EL **(**Fig. 1b). Consistent with a previous report^23^, we also found that phospholipase activity of the T111I mutant was similar to wild-type, whether using an artificial substrate **(**Fig. 1c), or when measuring hydrolysis of phospholipids of human HDL (Fig. 1d).

### Stability and structure of EL T111I

**Figure 2:**
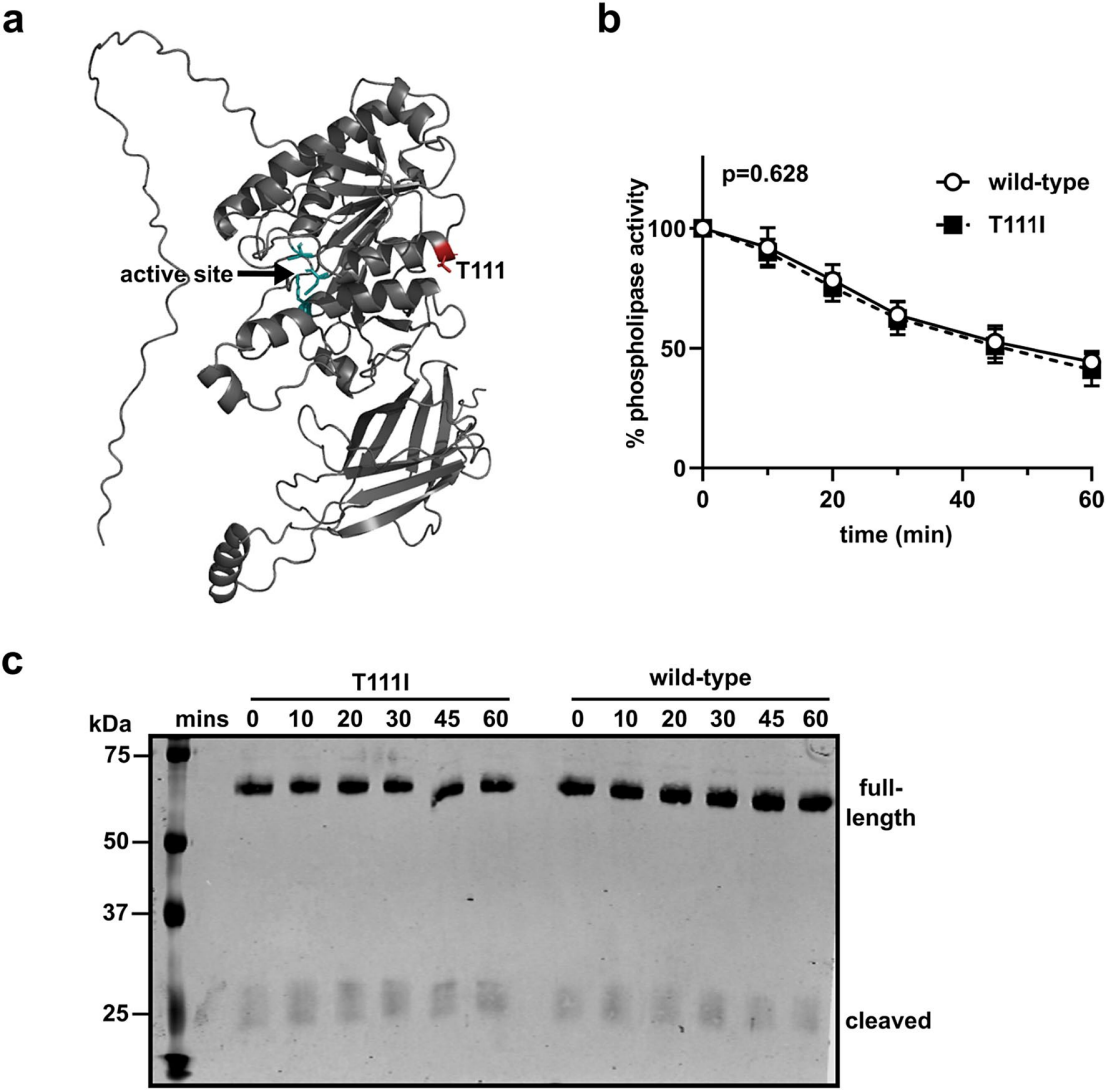
Stability of EL T111I. (**a**) Structure of WT EL as predicted by ColabFold. The catalytic triad of EL is depicted in green and the residue T111 is colored in red. (**b**) Activity over time of WT and T111I EL conditioned media incubated at 37 °C. Activity at each time point was measured using a fluorescence-based phospholipase activity assay. Points represents 5 independent experiments (mean ± SD). P value determined by one phase decay least squares fit. (**c**) Western of T111I and wild-type EL after incubation at 37 °C with increasing time. Uncropped blot shown in Supplemental Fig. 1a.

The structure of EL has not been solved. We utilized ColabFold^33^, a derivative of Alphafold2^34^, to predict the structure of wild-type human EL (Fig. 2a). In agreement with previously published predictions^35^, the T111 residue is located at the end of an alpha helix in the N-terminus domain of EL. In tandem with the predicted structure, we utilized Dynamut2^36^ to predict how the single mutation, T111I, might alter the stability of EL. Using Dynamut2 we calculated the ΔΔG of the T111I mutation using the top 5 predicted EL structures as outputted by ColabFold. The predicted ΔΔG^Stability^ was − 0.18 kcal/mol for the top ranked model (the model shown in Fig. 2a). The range for all 5 models was − 0.39 kcal/mol to − 0.14 kcal/mol. When we used Dynamut2 to calculate the ΔΔG^Stability^ of WT EL as determined by AlphaFold2 (AF-Q9Y5X9-F1), the ΔΔG^Stability^ was − 0.33 kcal/mol. These values indicate that the T111I mutation could potentially be mildly destabilizing^37^.We have previously observed that wild-type EL spontaneously loses activity at 37 °C^38^, a trait shared with the closely related lipase, lipoprotein lipase^39,40^. Therefore, we asked if the T111I EL spontaneously lost activity at a different rate than wild-type EL. However, we found that, at 37 °C, EL with the T111I mutation lost activity at a similar rate compared to wild-type (Fig. 2b). The loss of activity for both WT and T111I EL did not appear to be mediated by proteolysis as there was no evidence of novel cleavage or increased degradation over time in these samples (Fig. 2c).

### Inhibition of EL T111I by angiopoietin-like proteins

EL shares 44% homology with the closely related lipase, lipoprotein lipase (LPL). LPL is inhibited by both ANGPTL3, in the form of ANGPTL3/8 complexes, and by ANGPTL4^26–28,40–48^. The interaction between LPL and ANGPTL4 has been extensively studied and potential binding sites between the two proteins have been mapped through deuterium exchange studies^39,49^.

**Figure 3:**
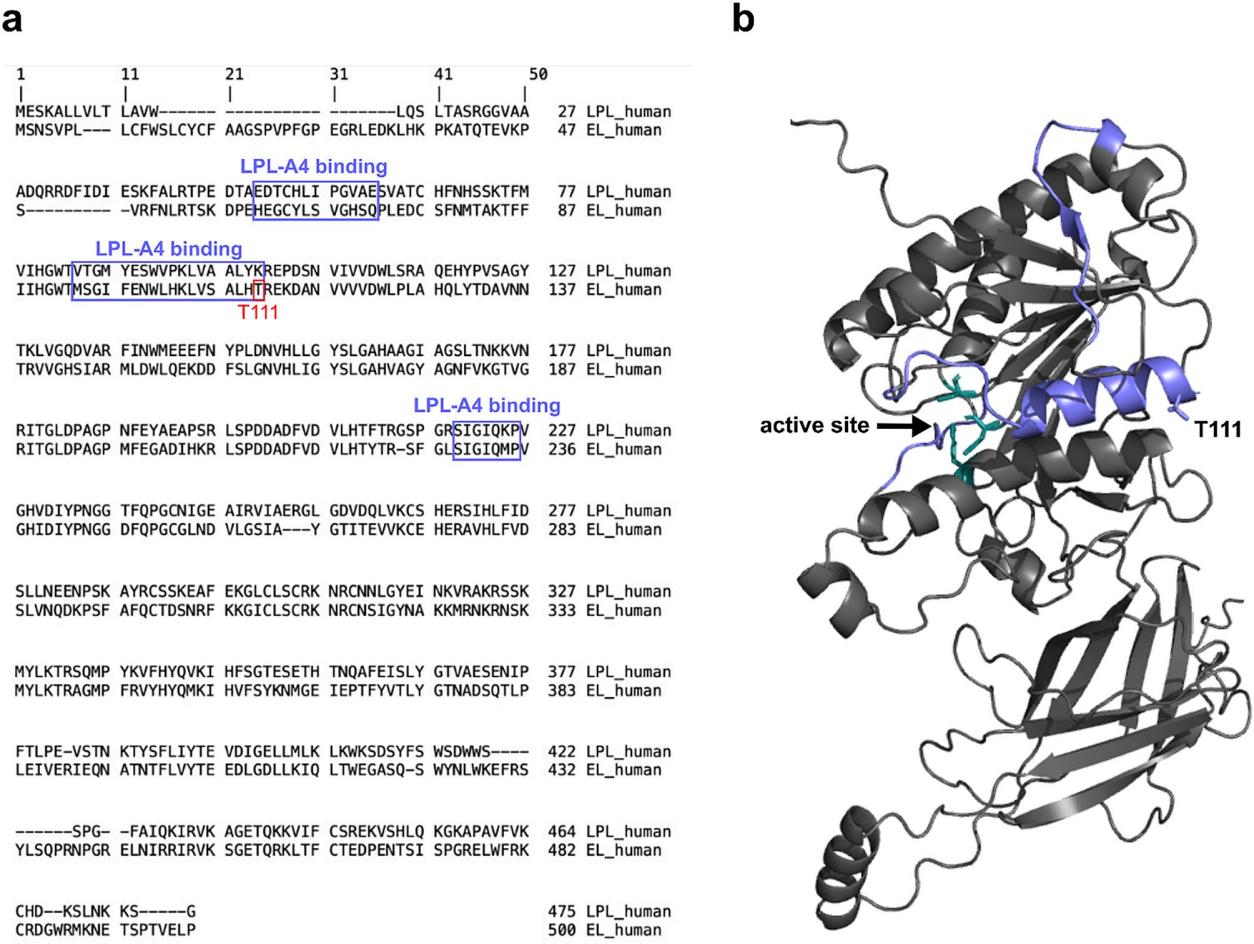
Potential ANGPTL binding sites on EL. (**a**) MAFFT multiple sequence alignment of human LPL and human EL. ANGPTL4 binding sites on LPL and the homologous regions of EL are boxed in purple. The T111 residue is boxed in red. (**b**) Predicted structure of WT EL indicating the catalytic triad (green) and the regions homologous to the ANGPTL4 binding sites on LPL (purple).

**Figure 4:**
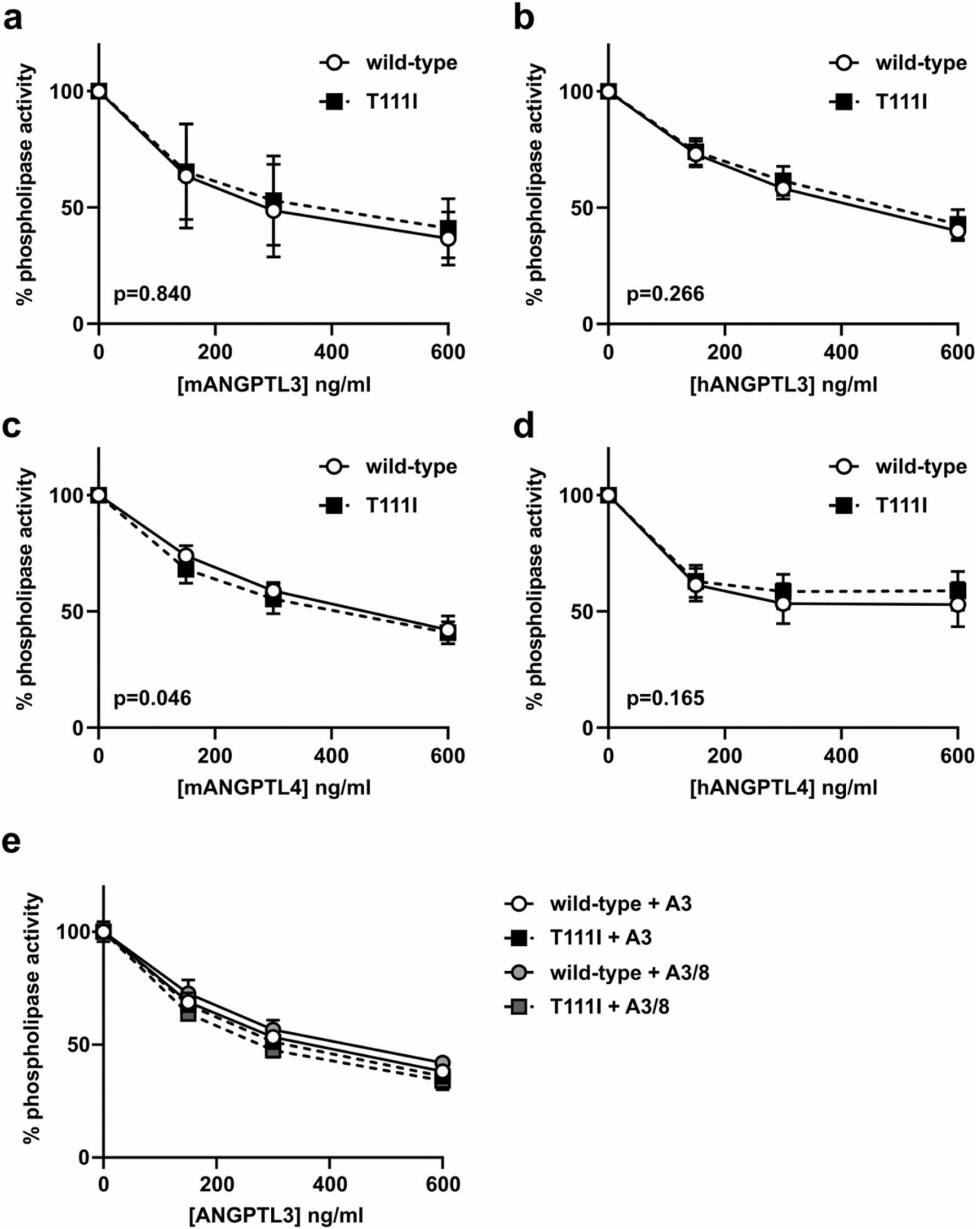
Inhibition of EL T111I by ANGPTL3 and ANGPTL4. WT EL and T111I EL were incubated at 37 °C for 30 min with increasing concentrations of (**a**) mouse ANGPTL3, (**b**) human ANGPTL3, (**c**) mouse ANGPTL4, (**d**) human ANGPTL4, or (**e**) mouse ANGPTL3-ANGPTL8 complexes. Activity was measured using a fluorescence-based phospholipase activity assay. Points represent mean (± SD) of 4 experiments each run with 2 biological duplicates. P values determined by one phase decay least squares fit.

Because EL is closely related to LPL, we asked if the T111 mapped to an area homologous to an ANGPTL4 binding domain in LPL. We aligned the protein sequence of human EL against human LPL using the Clustal format alignment by MAFFT^50^ (Fig. 3a). When comparing homology between EL and LPL, the T111 residue aligns with one of the binding site clusters to which ANGPTL4 binds (Fig. 3a,b). Therefore, it is possible that ANGPTL4 and/or ANGPTL3 interacts with EL at the same site, and that the T111I mutation could disrupt this interaction. To test this idea, we first measured EL phospholipase activity after incubation with increasing concentrations of human or mouse ANGPTL3 protein. Inhibition of both wildtype and T111I EL was dose-dependent, consistent with our previous findings^38^. Moreover, both human and mouse ANGPTL3 inhibited T111I EL to a similar level as wild-type EL **(**Fig. 4a,b**)**. Recently ANGPTL4 has also been reported to inhibit EL^51^. Therefore, we asked if ANGPTL4 inhibition was affected by the T111I mutation. As with ANGPTL3, there was little difference in inhibition of T111I EL by ANGPTL4 when compared to wild-type EL **(**Fig. 4c,d**)**. Although we previously showed that ANGPTL8 did not affect the ability of ANGPTL3 to inhibit EL^38^, we considered the possibility that ANGPTL8 would have an effect on T111I EL. However, we saw little difference in the inhibition of T111I EL when comparing ANGPTL3 and ANGPTL3 with ANGPTL8 (Fig. 4e).

**Figure 5:**
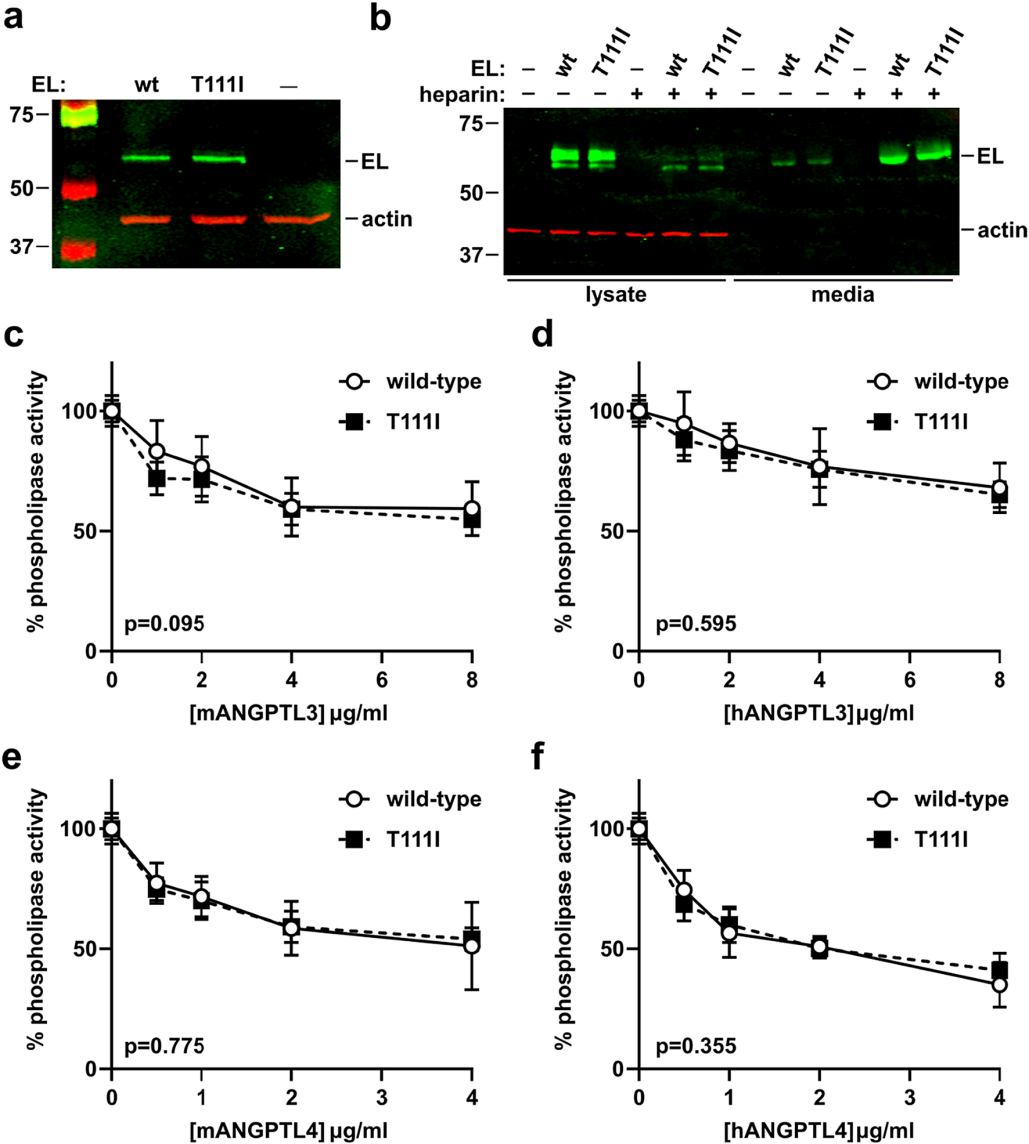
Inhibition of membrane-tethered EL T111I by ANGPTL3 and ANGPTL4. (**a**) Expression of WT and T111I EL from transduced RHMVEC. Western blot shows expression of EL in the lysate of virally transduced endothelial cells. (**b**) Release of HSPG-bound wild-type and T111I EL from the cell membrane. Western blot shows EL in lysate and media of wild-type and T111I expressing cells treated with ± 10 U/mL heparin for 24 h. Uncropped blots shown in Supplemental Fig. 1b,c. (**c**–**f**) WT EL and T111I EL expressing RHMVEC were incubated at 37 °C for 30 min with increasing concentrations of (**c**) mouse ANGPTL3, (**d**) human ANGPTL3, (**e**) mouse ANGPTL4, or (**f**) human ANGPTL4. Activity was measured using a fluorescence-based phospholipase activity assay. Points represent mean (± SD) of 4 experiments each run with 2 biological duplicates. P values determined by one phase decay least squares fit.

In vivo, EL can be bound to the surface of endothelial cells through interactions with heparan sulfate proteoglycans (HSPGs)^52^, and we have previously found that EL bound to endothelial cells is resistant to ANGPTL3 inhibition^38^. We therefore asked if the T111I substitution affected the ability of endothelial cell-bound EL to be inhibited by ANGPTL3 or ANGPTL4. We stably expressed WT EL or T111I in rat heart microvessel endothelial cells (RHMVECs) (Fig. 5a). To evaluate if the EL protein produced by these cell lines was properly secreted and bound to the outside of the cell, we treated EL expressing cells with 10 U/mL heparin to release HSPG-bound EL from the membrane. We found that the majority of both WT and T111I EL was released into the media after heparin treatment, suggesting appropriate membrane localization (Fig. 5b). To test inhibition of membrane-bound EL, we treated WT and T111I EL expressing RHMVECs with ANGPTL3 or ANGPTL4 and measured phospholipase activity. As expected, EL bound to the endothelial cell surface was resistant to ANGPTL3 and ANGPTL4 inhibition (Fig. 5). However, we again found little difference between WT and T111I EL as far as inhibition by ANGPTL3 (Fig. 5c,d) or ANGPTL4 (Fig. 5e,f).

## Discussion

In this study, we evaluated a common EL variant, T111I, for its effects on EL activity, stability, and ability to be inhibited by ANGPTL proteins. In all these parameters, we found that EL T111I was no different than wildtype EL.

The 584C/T polymorphism, which encodes the T111I mutation in EL, has been associated with increased HDL levels in some population studies. The variant was originally discovered by sequencing black and Caucasian individuals with high HDL-C levels^11^. Subsequently, several groups found an association between the T111I variant and HDL-C levels and other cardiovascular risk factors^10,12–17^, but several other studies found no association^18–23^. Numerous studies continue to be performed to evaluate a potential association between this EL variant, HDL-C levels, and the risk of CAD. A meta-analysis, performed by Cai et al*.* in 2014, included 9 case–control studies of Caucasian and Asian populations. This meta-analysis, which considered the sample size of the previously published studies and repeated the statistical analysis, found that carriers of the 584C/T polymorphism did have higher HDL-C levels compared to non-carriers but did not find a significant association between the variant and the risk of coronary heart disease^17^. A second meta-analysis performed by Zhao et al*.* in 2020 included 13 studies from countries including The United States, China, Japan, Netherlands, Iran, Egypt, and Turkey. This analysis did not examine association with HDL-C levels, but did find that EL 584C/T was associated with CAD susceptibility^53^. A third meta-analysis by Wu et al. included 14 case–control studies with 9731 subjects^54^. This meta-analysis, which again did not analyze HDL-C levels, also found a significant association between the EL 584C/T polymorphism and CAD, but only in African populations^54^. It is important to note, however, that the authors of this study clearly disclosed that only one of the fourteen eligible studies could be analyzed for the African populations. Thus, although an association may be possible between T111I and HDL-C or CAD for different ethnic groups, the lack of eligible studies may influence the ability to confidently draw conclusions. Moreover, even if the T111I allele is associated with increased cardiovascular risk, it may not be causal, with causality coming from other alleles in the same haplotype^22,35^. In summary, the association between the EL T111I variant and HDL-C or the risk of CAD remains somewhat unsettled, and there is, to date, no evidence of causality.

If T111I EL is indeed associated with high HDL levels, the simplest explanation is that it alters EL function. Functional changes could include changes in enzymatic activity, changes in stability, and changes in interactions with other proteins, such as the endogenous inhibitor ANGPTL3. A previous study by Edmonson et al. found no difference in phospholipase activity between wild-type and T111I EL when tested either by measuring the hydrolysis of dipalmitoylphosphatidyl choline or by using isolated human HDL_3_ as a substrate^23^. We likewise found no differences in phospholipase activity using either a fluorescence-based assay or fatty-acid release from human HDL particles.

Although the structure of EL has not been solved experimentally, the ColabFold structure puts the T111 at the end of an alpha helix in the N-terminal domain of EL. Although the N-terminal domain contains the catalytic domain, T111 is not near the active site, nor does it seem positioned to contribute to proper folding of EL. We found no evidence that the T111I mutation disrupts EL stability, at least in vitro. These data support previous predictions which utilized homology modelling to predict the structure of EL and CHARMM calculation protocols to predict stability changes and suggested that the T111I mutation does not have any significant effects on the EL protein stability^35^.

The location of T111 on the outer face of an alpha helix on EL suggests that it could potentially be involved in interactions with other proteins. Indeed, in the homologous lipase, LPL, this same alpha helix is thought to interact with the lipase inhibitor ANGPTL4^39,49^. However, it is important to note that T111 is not conserved between EL and LPL. Moreover, we did not observe any meaningful differences in inhibition by either ANGPTL3 or ANGPTL4 when threonine 111 was mutated to isoleucine. We conclude that if T111 is involved in protein binding, it is not with ANGPTL proteins.

The data in this paper suggest that the T111I variation does not alter EL specific activity, protein stability, or inhibition by ANGPTL proteins. However, it is important to note that it is also possible that T111I induces small enough changes in EL activity, stability, or inhibition by ANGPTL proteins that the sensitivity and reproducibility of our assays were unable to detect these differences. Moreover, our findings do not rule out the possibility that T111I alters or compromises EL function in other ways that could potentially alter HDL levels or function. The T111I variant introduces a large hydrophobic residue on the surface of EL, which would increase the propensity for non-specific hydrophobic associations or could alter the interactions of EL with proteins other than ANGPTLs. This change in hydrophobicity could also change the identity and specificity of lipoprotein binding to EL. T111I EL might bind preferentially to different sizes of HDL compared to wildtype EL or bind more strongly to non-HDL lipoproteins thus interfering with HDL binding. Such changes could also alter the bridging function of EL^52,55^. Subtle changes in EL structure cause by the T111I variant could also alter post-translational modifications or substrate specificity. Any of these changes could alter EL function in vivo. An alteration in substrate specificity, for example, could result in changes in HDL lipid composition and subsequent changes in the proteome and functionality of HDL. These possibilities could be explored by using mass spectrometry to examine the lipid and protein composition of HDL after incubation with wildtype or T111I EL. HDL functions, including cholesterol efflux, paraoxonase activity, and anti-inflammation could also be assayed following EL incubation. It is unclear, however, given the uncertainty of the connection between T111I and any physiological changes, how much continued effort should be put into pursuing possible differences between wild-type and T111I EL.

## Methods

### Expression constructs

The generation of plasmid constructs expressing strep-tagged mouse ANGPTL3 (pHS18), strep-tagged human ANGPTL3 (pHS15), and V5-tagged mouse ANGPTL8 (pWL1) have been described previously^26^. The generation of the plasmid construct expression V5-tagged human ANGPTL4 (pHS2) has been described previously^56^.

A plasmid expressing untagged human EL (pKS6) was generated by amplifying the coding sequence of human EL from cDNA (LIPG; MGC MHS6278-202806078) and inserting it into the vector pCDNA6 using InFusion cloning (Clontech). An EL containing a T111I mutation (pKS17) was generated by introducing a *332C* > *T* substitution into the EL coding region of pKS6 using site-directed mutagenesis. A lentiviral construct expressing EL with a T111I mutation (pKS18) was generated by introducing a *332C* > *T* substitution into the EL coding region of pKS7, a lentiviral construct expressing wild-type EL^38^. Lentiviruses with this construct were produced by transfecting human embryonic kidney 293 T (HEK 293 T) cells with pKS18 and the lentiviral packaging vectors pMD2.G (Addgene plasmid #12259; http://n2t.net/addgene:12259; RRID:Addgene 12259), pRSV-Rev (Addgene plasmid #12253; http://n2t.net/addgene:12253; RRID:Addgene 12253)^57^, and pMDLg/pRRE (Addgene plasmid #12251; http://n2t.net/addgene:12251; RRID:Addgene 12251)^57^. All lentiviral packaging vectors were gifts from Didier Trono. Lentivirus-containing media was collected, concentrated using Lenti-X Concentrator (Clontech; catalog no. 631231), and used to generate EL-expressing cell lines as described below. A construct expressing full-length V5 tagged mouse ANGPTL4 (pHS5) was generated by amplifying full-length mouse ANGPTL4 cDNA (OpenBiosystems) and inserting it into a pCDNA6 vector using In-Fusion cloning (Clontech). A V5 tag was then appended to the C terminus of the open reading frame using Phusion site-directed mutagenesis (New England Biolabs).

### Cell lines

HEK 293 T cells (ATCC) were grown and maintained in DMEM supplemented with 5% FBS, 1% penicillin/streptomycin antibiotics, and 1% L-Glutamine (complete DMEM).

Rat heart microvessel endothelial cells (RHMVECs; VEC Technologies, Inc) were grown in MCDB-131 base medium (GenDEPOT) supplemented with 10 mM L-glutamine, 1% penicillin/streptomycin antibiotic solution (10,000 U/ml penicillin and 10,000 μg/ml streptomycin; Gibco), 5% fetal bovine serum (Atlanta Biologicals), 1 μg/ml hydrocortisone (Sigma-Aldrich), 10 μg/ml human epidermal growth factor (Gibco, Life Technologies), and 12 μg/ml bovine brain extract (Lonza). To generate a stable cell line expressing human EL (T111I), 80% confluent RHMVECs in 6-well plates were transduced with human T111I EL (pKS18) lentiviruses with 4 μg/ml Polybrene (#134220; Santa Cruz Biotechnology) in a total volume of 1 ml DMEM. Twenty-four hours post-transduction, cells were washed with PBS and incubated in MCDB-131 complete medium for 48 h. Cells were then subjected to selection with 5 μg/ml puromycin for 5 days. RHMVECs stably expressing wild-type human EL (pKS7-RHMVECs) were generated as described previously^38^. To test the proper membrane localization of EL in these cell lines, RHMVECs stably expressing wild-type human EL (pKS7-RHMVECs), T111I human EL (pKS18-RHMVECs), or untransduced RHMVECs were grown to confluency in fibronectin-coated T25 flasks. Media was changed to serum-free OptiMEM containing 1X protease arrest and ± 10 U/mL heparin (Fresenius Kabi USA, LLC). After 24 h, media and lysate were collected. Cells were washed with PBS and lysed in RIPA buffer containing 1X protease arrest. Presence of EL in the lysate and media was measured by western blot.

### Production and quantification of ANGPTL3, ANGPTL4, and ANGPTL3 + 8 conditioned media

To produce human and mouse ANGPTL3 and ANGPTL4 protein, HEK 293 T cells were grown to 80% confluency in T25 flasks in complete DMEM. Cells were transfected with 5 μg of pHS18 (strep-tagged mouse ANGPTL3), pHS15 (strep-tagged human ANGPTL3), pHS5 (V5-tagged mouse ANGPTL4), or pHS2 (V5-tagged human ANGPTL4) and 10 μl of 1 mg/ml PEI (polyethylenimine). To produce mouse ANGPTL3 and ANGPTL8 complexes, HEK 293 T were grown to 80% confluency in T75 flasks and transfected with 5 μg of pHS18 and 5 μg of pWL1 (V5-tagged mouse ANGPTL8) simultaneously. Cells were switched to serum-free DMEM and 1X ProteaseArrest protease inhibitor cocktail (APExBIO) 24 h post-transfection. Conditioned media were collected 48–72 h later. To produce 293 T control media (CM), HEK293T cells were transfected and media collected in the same manner, only no DNA was added to the transfection. This control conditioned media was used as a control for all experiments involving ANGPTL conditioned media. The concentration of ANGPTL3 in conditioned media was determined via western quantification against a batch of mouse ANGPTL3 with a known concentration, previously quantified via ELISA kit (RayBiotech, catalog #ELM-ANGPTL3-1). The concentration of ANGPTL4 in conditioned media was determined via western quantification against a batch of human ANGPTL4 with a known concentration, previously quantified via a human ANGPTL4 ELISA kit (Sigma).

### Production and quantification of endothelial lipase conditioned media

To produce EL conditioned media, HEK 293 T were grown to 80% confluency in DMEM supplemented with 5% FBS, Penicillin/streptomycin antibiotics, and L-Glutamine. Cells were transfected with 10 μg of pKS6 (human WT EL) or pKS17 (human T111I EL) and 20 μl of 1 mg/ml PEI (polyethylenimine). Media was changed to serum-free OptiMEM containing 1X ProteaseArrest (APExBIO) and 0.1 U/ml heparin (Fresenius Kabi USA, LLC) 24 h post-transfection. EL conditioned media was collected after an additional 24 h. Protein expression was confirmed by western blotting and tested via phospholipase activity assay. EL concentration was determined by comparing EL protein signal with a quantified batch of strep-tagged human EL protein via quantitative western blot.

### Western blot

Protein samples were size fractionated on 12% SDS-PAGE gels and then transferred to a nitrocellulose membrane. Membranes were blocked with casein buffer (1% casein, Fisher Science Education). Primary antibodies were diluted in casein buffer + 0.1% Tween. Primary antibody dilutions were 1:3000 for a mouse monoclonal antibody against EL (LIPG antibody clone 3C7, Lifespan Biosciences), 1:2000 for a goat antibody against beta-actin (Abcam), 1:3000 for a rabbit polyclonal antibody against Strep-tag II (Abcam) to detect Strep-tagged ANGPTL3, or 1:6000 for a mouse monoclonal antibody against V5 tag (R960-25; Invitrogen) to detect V5-tagged ANGPTL4. After washing with PBS-T, membranes were incubated with Dylight680- or Dylight800-labeled secondary antibodies (Thermo Scientific) diluted 1:5000 in casein. After washing with PBS-T, antibody binding was detected using an Odyssey Clx Infrared Scanner (Li-Cor).

### EL activity assays

Phospholipase activity was monitored using the EnzChek Phospholipase A1 assay kit (ThermoFisher) as described previously^58^. EL and ANGPTL3 or ANGPTL4 proteins in conditioned media were combined and incubated at 37 °C for 30 min. Following incubation, 50 μl of sample was mixed with 50 μl of substrate solution and were incubated at room temperature (approximately 20–22 °C) for 30 min, reading fluorescence (485 nm excitation/515 nm emission) every 1 or 2 min with an Infinite F200 plate reader (Tecan). Relative phospholipase activity was calculated by calculating the slope of the linear part of the curve (typically in the range between 5 to 25 min) and then subtracting out the slope of the blank (sample with no EL conditioned media).

To detect phospholipase activity on the cell surface, RHMVECs expressing WT EL (pKS7-RHMVEC) or T111I EL (pKS18-RHMVEC) were grown to confluency in 96-well clear-bottom plates coated with fibronectin. After washing twice with 1 × sterile PBS, cells were incubated with 50 μl of ANGPTL3, ANGPTL4, or control conditioned media for 30 min at 37 °C. After incubation, 50 μl of EnzChek Phospholipase A1 assay substrate was added, and fluorescence (485 nm excitation/515 nm emission) was read at room temperature (approximately 20–22 °C) every 2 min for 30 min with an Infinite F200 plate reader. Relative phospholipase activity was determined by calculating the slope of the linear part of the curve (typically in the range between 5 and 25 min) and then subtracting out the slope of samples containing untransduced RHMVECs incubated with 50 μl control conditioned media. On average the phospholipase activity from untransduced RHMVECs was ~ 21% of that of EL transduced RHMVECs (20.7 with standard deviation of 9.96). Each condition was normalized as a percentage of their respective positive control (either WT or T111I EL expressing cells which did not receive any ANGPTL conditioned media).

### EL activity assay with HDL substrate

Human HDL (a kind gift from Dr. Kasey Vickers, prepared as described previously^59^) at a final concentration of 1.136 mg/ml was incubated with EL conditioned media for 60 min at 37 °C. After incubation, 50 μl of sample was loaded into a Greiner clear 96-well plate. NEFA levels in each sample were measured using a commercial kit (HR series NEFA-HR, Wako) and following the manufacturer’s instructions. Briefly, 112.5 μl of NEFA Reagent A (0.53 U/ml Acyl-coenzyme A synthetase, 0.31 mmol Coenzyme A, 4.3 mmol/L Adenosine triphosphate, 1.5 mmol/L 4-aminoanitipyrine, 2.6 U/ml Ascorbate oxidase, 0.062% Sodium azide) was added to each sample and incubated for 10 min at 37 °C. 37.5 μl of NEFA Reagent B (12 U/ml Acyl-coenzyme A oxidase, 14 U/ml Perosidase) was then added and samples were incubated for 5 min at 37 °C. Absorbance was read at 550 and 650 nm with an Infinite F200 Pro plate reader (Tecan). For data analysis, the values read at 650 nm were subtracted from 550 nm values.

### Predicted structure modeling

The protein structure was predicted using ColabFold^33^, an adaptation of AlphaFold2^34^ which uses a slightly different algorithm to generate the multiple sequence alignment. The EL FASTA sequence was acquired from the Uniprot database entry Q9Y5X9^60^. This sequence was used as an input to generate the MSA used by the AlphaFold2 algorithm. Five predicted structures were generated from the algorithm and the structure with the highest confidence rank was selected to be the representative structure. Figures were generated using Pymol by importing the pdb files generated from ColabFold.

### Statistics

Statistics were performed using GraphPad Prism 9. Single point lipase activity assays comparing EL and EL T111I were analyzed using unpaired student *t* tests. All dose–response and time-course activity curves were compared using one phase decay least squares fit.

### Supplementary Information

Supplementary Figure 1.

## Data Availability

All data generated or analyzed during this study are included in this published article (and its Supplementary Information files).

## References

[1] Tall AR (1998). An overview of reverse cholesterol transport. Eur. Heart J..

[2] Wang N, Lan D, Chen W, Matsuura F, Tall AR (2004). ATP-binding cassette transporters G1 and G4 mediate cellular cholesterol efflux to high-density lipoproteins. Proc. Natl. Acad. Sci. U.S.A..

[3] Larrede S, Quinn CM, Jessup W, Frisdal E, Olivier M, Hsieh V, Kim M-J, Van Eck M, Couvert P, Carrie A, Giral P, Chapman MJ, Guerin M, Le Goff W (2009). Stimulation of cholesterol efflux by LXR agonists in cholesterol-loaded human macrophages is ABCA1-dependent but ABCG1-independent. Arterioscler. Thromb. Vasc. Biol..

[4] Tarling EJ, Edwards PA (2011). ATP binding cassette transporter G1 (ABCG1) is an intracellular sterol transporter. Proc. Natl. Acad. Sci. U.S.A..

[5] Bowry VW, Stanley KK, Stocker R (1992). High density lipoprotein is the major carrier of lipid hydroperoxides in human blood plasma from fasting donors. Proc. Natl. Acad. Sci. U.S.A..

[6] Hirata K, Dichek HL, Cioffi JA, Choi SY, Leeper NJ, Quintana L, Kronmal GS, Cooper AD, Quertermous T (1999). Cloning of a unique lipase from endothelial cells extends the lipase gene family. J. Biol. Chem..

[7] Jahangiri A, Rader DJ, Marchadier D, Curtiss LK, Bonnet DJ, Rye K-A (2005). Evidence that endothelial lipase remodels high density lipoproteins without mediating the dissociation of apolipoprotein A-I. J. Lipid Res..

[8] Jaye M, Lynch KJ, Krawiec J, Marchadier D, Maugeais C, Doan K, South V, Amin D, Perrone M, Rader DJ (1999). A novel endothelial-derived lipase that modulates HDL metabolism. Nat. Genet..

[9] Ishida T, Choi S, Kundu RK, Hirata K, Rubin EM, Cooper AD, Quertermous T (2003). Endothelial lipase is a major determinant of HDL level. J. Clin. Investig..

[10] Ma K, Cilingiroglu M, Otvos JD, Ballantyne CM, Marian AJ, Chan L (2003). Endothelial lipase is a major genetic determinant for high-density lipoprotein concentration, structure, and metabolism. Proc. Natl. Acad. Sci..

[11] deLemos AS, Wolfe ML, Long CJ, Sivapackianathan R, Rader DJ (2002). Identification of genetic variants in endothelial lipase in persons with elevated high-density lipoprotein cholesterol. Circulation.

[12] Mank-Seymour AR, Durham KL, Thompson JF, Seymour AB, Milos PM (2004). Association between single-nucleotide polymorphisms in the endothelial lipase (LIPG) gene and high-density lipoprotein cholesterol levels. Biochim. Biophys. Acta.

[13] Hutter CM, Austin MA, Farin FM, Viernes H-M, Edwards KL, Leonetti DL, McNeely MJ, Fujimoto WY (2006). Association of endothelial lipase gene (LIPG) haplotypes with high-density lipoprotein cholesterol subfractions and apolipoprotein AI plasma levels in Japanese Americans. Atherosclerosis.

[14] Tang N-P, Wang L-S, Yang L, Zhou B, Gu H-J, Sun Q-M, Cong R-H, Zhu H-J, Wang B (2008). Protective effect of an endothelial lipase gene variant on coronary artery disease in a Chinese population. J. Lipid Res..

[15] Paradis M-E, Couture P, Bosse Y, Despres J-P, Perusse L, Bouchard C, Vohl M-C, Lamarche B (2003). The T111I mutation in the EL gene modulates the impact of dietary fat on the HDL profile in women. J. Lipid Res..

[16] Solim LA, Gencan IA, Çelik B, Ataacar A, Koç U, Büyükören B, Güngör G, Isbir S (2018). Endothelial lipase gene polymorphism (584 C/T) in coronary artery patients among a Turkish population. In Vivo.

[17] Cai G, Huang Z, Zhang B, Weng W, Shi G (2014). The associations between endothelial lipase 584C/T polymorphism and HDL-C level and coronary heart disease susceptibility: A meta-analysis. Lipids Health Dis..

[18] Halverstadt A, Phares DA, Ferrell RE, Wilund KR, Goldberg AP, Hagberg JM (2003). High-density lipoprotein-cholesterol, its subfractions, and responses to exercise training are dependent on endothelial lipase genotype. Metabolism.

[19] Shimizu M, Kanazawa K, Hirata K, Ishida T, Hiraoka E, Matsuda Y, Iwai C, Miyamoto Y, Hashimoto M, Kajiya T, Akita H, Yokoyama M (2007). Endothelial lipase gene polymorphism is associated with acute myocardial infarction, independently of high-density lipoprotein-cholesterol levels. Circ. J..

[20] Jensen MK, Rimm EB, Mukamal KJ, Edmondson AC, Rader DJ, Vogel U, Tjønneland A, Sørensen TIA, Schmidt EB, Overvad K (2009). The T111I variant in the endothelial lipase gene and risk of coronary heart disease in three independent populations. Eur. Heart J..

[21] Yamakawa-Kobayashi K, Yanagi H, Endo K, Arinami T, Hamaguchi H (2003). Relationship between serum HDL-C levels and common genetic variants of the endothelial lipase gene in Japanese school-aged children. Hum. Genet..

[22] Razzaghi H, Santorico SA, Kamboh MI (2012). Population-based resequencing of LIPG and ZNF202 genes in subjects with extreme HDL levels. Front. Genet..

[23] Edmondson AC, Brown RJ, Kathiresan S, Cupples LA, Demissie S, Manning AK, Jensen MK, Rimm EB, Wang J, Rodrigues A, Bamba V, Khetarpal SA, Wolfe ML, DerOhannessian S, Li M, Reilly MP, Aberle J, Evans D, Hegele RA, Rader DJ (2009). Loss-of-function variants in endothelial lipase are a cause of elevated HDL cholesterol in humans. J. Clin. Investig..

[24] Smith CE, Arnett DK, Tsai MY, Lai C-Q, Parnell LD, Shen J, Laclaustra M, Junyent M, Ordovás JM (2009). Physical inactivity interacts with an endothelial lipase polymorphism to modulate high density lipoprotein cholesterol in the GOLDN study. Atherosclerosis.

[25] Shimamura M, Matsuda M, Yasumo H, Okazaki M, Fujimoto K, Kono K, Shimizugawa T, Ando Y, Koishi R, Kohama T (2007). Angiopoietin-like protein3 regulates plasma HDL cholesterol through suppression of endothelial lipase. Arterioscler. Thromb. Vasc. Biol..

[26] Chi X, Britt EC, Shows HW, Hjelmaas AJ, Shetty SK, Cushing EM, Li W, Dou A, Zhang R, Davies BSJ (2017). ANGPTL8 promotes the ability of ANGPTL3 to bind and inhibit lipoprotein lipase. Mol. Metab..

[27] Lee E-C, Desai U, Gololobov G, Hong S, Feng X, Yu X-C, Gay J, Wilganowski N, Gao C, Du L-L, Chen J, Hu Y, Zhao S, Kirkpatrick L, Schneider M, Zambrowicz BP, Landes G, Powell DR, Sonnenburg WK (2009). Identification of a new functional domain in angiopoietin-like 3 (ANGPTL3) and angiopoietin-like 4 (ANGPTL4) involved in binding and inhibition of lipoprotein lipase (LPL). J. Biol. Chem..

[28] Shimizugawa T, Ono M, Shimamura M, Yoshida K, Ando Y, Koishi R, Ueda K, Inaba T, Minekura H, Kohama T, Furukawa H (2002). ANGPTL3 decreases very low density lipoprotein triglyceride clearance by inhibition of lipoprotein lipase. J. Biol. Chem..

[29] Shan L, Yu X-C, Liu Z, Hu Y, Sturgis LT, Miranda ML, Liu Q (2009). The angiopoietin-like proteins ANGPTL3 and ANGPTL4 inhibit lipoprotein lipase activity through distinct mechanisms. J. Biol. Chem..

[30] Sonnenburg WK, Yu D, Lee E-C, Xiong W, Gololobov G, Key B, Gay J, Wilganowski N, Hu Y, Zhao S, Schneider M, Ding Z-M, Zambrowicz BP, Landes G, Powell DR, Desai U (2009). GPIHBP1 stabilizes lipoprotein lipase and prevents its inhibition by angiopoietin-like 3 and angiopoietin-like 4. J. Lipid Res..

[31] Yau M, Wang Y, Lam KSL, Zhang J, Wu D, Xu A (2009). A highly conserved motif within the NH2-terminal coiled-coil domain of angiopoietin-like protein 4 confers its inhibitory effects on lipoprotein lipase by disrupting the enzyme dimerization. J. Biol. Chem..

[32] Jin W, Wang X, Millar JS, Quertermous T, Rothblat GH, Glick JM, Rader DJ (2007). Hepatic proprotein convertases modulate HDL metabolism. Cell Metab..

[33] Mirdita, M., K. Schütze, Y. Moriwaki, L. Heo, S. Ovchinnikov, and M. Steinegger. ColabFold - Making protein folding accessible to all. https://www.biorxiv.org/content/10.1101/2021.08.15.456425v2 (Accessed 26 November 2021) (2021). 10.1038/s41592-022-01488-1 PMC9184281 35637307

[34] Jumper J, Evans R, Pritzel A, Green T, Figurnov M, Ronneberger O, Tunyasuvunakool K, Bates R, Žídek A, Potapenko A, Bridgland A, Meyer C, Kohl SAA, Ballard AJ, Cowie A, Romera-Paredes B, Nikolov S, Jain R, Adler J, Back T, Petersen S, Reiman D, Clancy E, Zielinski M, Steinegger M, Pacholska M, Berghammer T, Bodenstein S, Silver D, Vinyals O, Senior AW, Kavukcuoglu K, Kohli P, Hassabis D (2021). Highly accurate protein structure prediction with AlphaFold. Nature.

[35] Razzaghi H, Tempczyk-Russell A, Haubold K, Santorico SA, Shokati T, Christians U, Churchill MEA (2013). Genetic and structure-function studies of missense mutations in human endothelial lipase. PLoS One.

[36] Rodrigues CHM, Pires DEV, Ascher DB (2021). DynaMut2: Assessing changes in stability and flexibility upon single and multiple point missense mutations. Protein Sci..

[37] Rodrigues CHM, Portelli S, Ascher DB (2024). Exploring the effects of missense mutations on protein thermodynamics through structure-based approaches: Findings from the CAGI6 challenges. Hum. Genet..

[38] Sylvers-Davie KL, Segura-Roman A, Salvi AM, Schache KJ, Davies BSJ (2021). Angiopoietin-like 3 inhibition of endothelial lipase is not modulated by angiopoietin-like 8. J. Lipid Res..

[39] Leth-Espensen KZ, Kristensen KK, Kumari A, Winther A-ML, Young SG, Jørgensen TJD, Ploug M (2021). The intrinsic instability of the hydrolase domain of lipoprotein lipase facilitates its inactivation by ANGPTL4-catalyzed unfolding. Proc. Natl. Acad. Sci. U.S.A..

[40] Mysling S, Kristensen KK, Larsson M, Kovrov O, Bensadouen A, Jørgensen TJ, Olivecrona G, Young SG, Ploug M (2016). The angiopoietin-like protein ANGPTL4 catalyzes unfolding of the hydrolase domain in lipoprotein lipase and the endothelial membrane protein GPIHBP1 counteracts this unfolding. eLife.

[41] Ge H, Yang G, Yu X, Pourbahrami T, Li C (2004). Oligomerization state-dependent hyperlipidemic effect of angiopoietin-like protein 4. J. Lipid Res..

[42] Lichtenstein L, Mattijssen F, de Wit NJ, Georgiadi A, Hooiveld GJ, van der Meer R, He Y, Qi L, Köster A, Tamsma JT, Tan NS, Müller M, Kersten S (2010). Angptl4 protects against severe proinflammatory effects of saturated fat by inhibiting fatty acid uptake into mesenteric lymph node macrophages. Cell Metab..

[43] Mandard S, Zandbergen F, van Straten E, Wahli W, Kuipers F, Müller M, Kersten S (2006). The fasting-induced adipose factor/angiopoietin-like protein 4 is physically associated with lipoproteins and governs plasma lipid levels and adiposity. J. Biol. Chem..

[44] Köster A, Chao YB, Mosior M, Ford A, Gonzalez-DeWhitt PA, Hale JE, Li D, Qiu Y, Fraser CC, Yang DD, Heuer JG, Jaskunas SR, Eacho P (2005). Transgenic angiopoietin-like (angptl)4 overexpression and targeted disruption of angptl4 and angptl3: Regulation of triglyceride metabolism. Endocrinology.

[45] Sukonina V, Lookene A, Olivecrona T, Olivecrona G (2006). Angiopoietin-like protein 4 converts lipoprotein lipase to inactive monomers and modulates lipase activity in adipose tissue. Proc. Natl. Acad. Sci. U.S.A..

[46] Haller JF, Mintah IJ, Shihanian LM, Stevis P, Buckler D, Alexa-Braun CA, Kleiner S, Banfi S, Cohen JC, Hobbs HH, Yancopoulos GD, Murphy AJ, Gusarova V, Gromada J (2017). ANGPTL8 requires ANGPTL3 to inhibit lipoprotein lipase and plasma triglyceride clearance. J. Lipid Res..

[47] Kovrov O, Kristensen KK, Larsson E, Ploug M, Olivecrona G (2019). On the mechanism of angiopoietin-like protein 8 for control of lipoprotein lipase activity. J. Lipid Res..

[48] Chen YQ, Pottanat TG, Siegel RW, Ehsani M, Qian Y-W, Zhen EY, Regmi A, Roell WC, Guo H, Luo MJ, Gimeno RE, Van’t Hooft F, Konrad RJ (2020). Angiopoietin-like protein 8 differentially regulates ANGPTL3 and ANGPTL4 during postprandial partitioning of fatty acids. J. Lipid Res..

[49] Kristensen KK, Leth-Espensen KZ, Mertens HDT, Birrane G, Meiyappan M, Olivecrona G, Jørgensen TJD, Young SG, Ploug M (2020). Unfolding of monomeric lipoprotein lipase by ANGPTL4: Insight into the regulation of plasma triglyceride metabolism. Proc. Natl. Acad. Sci. U.S.A..

[50] Katoh K, Standley DM (2013). MAFFT multiple sequence alignment software version 7: Improvements in performance and usability. Mol. Biol. Evol..

[51] Chen YQ, Pottanat TG, Siegel RW, Ehsani M, Qian Y-W, Konrad RJ (2021). Angiopoietin-like protein 4 (ANGPTL4) is an inhibitor of endothelial lipase (EL) while the ANGPTL4/8 complex has reduced EL-inhibitory activity. Heliyon.

[52] Fuki IV, Blanchard N, Jin W, Marchadier DHL, Millar JS, Glick JM, Rader DJ (2003). Endogenously produced endothelial lipase enhances binding and cellular processing of plasma lipoproteins via heparan sulfate proteoglycan-mediated pathway. J. Biol. Chem..

[53] Zhao H, Zhao R, Hu S, Rong J (2020). Gene polymorphism associated with angiotensinogen (M235T), endothelial lipase (584C/T) and susceptibility to coronary artery disease: A meta-analysis. Biosci. Rep..

[54] Wu Y, Ma L, Zhang H, Chen X, Xu X, Hu Z (2020). Biosci. Rep..

[55] Strauss JG, Zimmermann R, Hrzenjak A, Zhou Y, Kratky D, Levak-Frank S, Kostner GM, Zechner R, Frank S (2002). Endothelial cell-derived lipase mediates uptake and binding of high-density lipoprotein (HDL) particles and the selective uptake of HDL-associated cholesterol esters independent of its enzymic activity. Biochem. J..

[56] Chi X, Shetty SK, Shows HW, Hjelmaas AJ, Malcolm EK, Davies BSJ (2015). Angiopoietin-like 4 modifies the interactions between lipoprotein lipase and its endothelial cell transporter GPIHBP1. J. Biol. Chem..

[57] Dull T, Zufferey R, Kelly M, Mandel RJ, Nguyen M, Trono D, Naldini L (1998). A third-generation lentivirus vector with a conditional packaging system. J. Virol..

[58] Basu D, Lei X, Josekutty J, Hussain MM, Jin W (2013). Measurement of the phospholipase activity of endothelial lipase in mouse plasma. J. Lipid Res..

[59] Michell DL, Allen RM, Landstreet SR, Zhao S, Toth CL, Sheng Q, Vickers KC (2016). Isolation of high-density lipoproteins for non-coding small RNA quantification. J. Vis. Exp..

[60] UniProt Consortium (2021). UniProt: The universal protein knowledgebase in 2021. Nucleic Acids Res..

