# Supplementary material for: Endothelial lipase variant T111I does not alter inhibition by angiopoietin-like proteins
Source: Sci Rep. 2024 Feb 20;14:4246. doi: 10.1038/s41598-024-54705-6 (PMC10879187; doi:10.1038/s41598-024-54705-6)
Supplement: Supplementary file 1 — Supplementary Figure 1. [file 41598_2024_54705_MOESM1_ESM.pdf]

Endothelial Lipase Variant T111I Does Not Alter Inhibition by Angiopoietin-like Proteins

Kelli L. Sylvers-Davie[1,2], Kaleb C. Bierstedt[1,3], Michael J. Schnieders[1,3], Brandon S. J. Davies[1,2*]

[1]Department of Biochemistry and Molecular Biology, [2]Fraternal Order of Eagles Diabetes Research Center, and [3]Department of Biomedical Engineering, University of Iowa, Iowa City, IA 52242

*Address Correspondence to: Brandon S. J. Davies, Department of Biochemistry and Molecular Biology, University of Iowa, 169 Newton Rd., PBDB 3326, Iowa City, IA, 52242. Tel.: 319-335-3225; Fax: 319-335-9570; E-mail: Brandon-davies@uiowa.edu

Supplemental Data

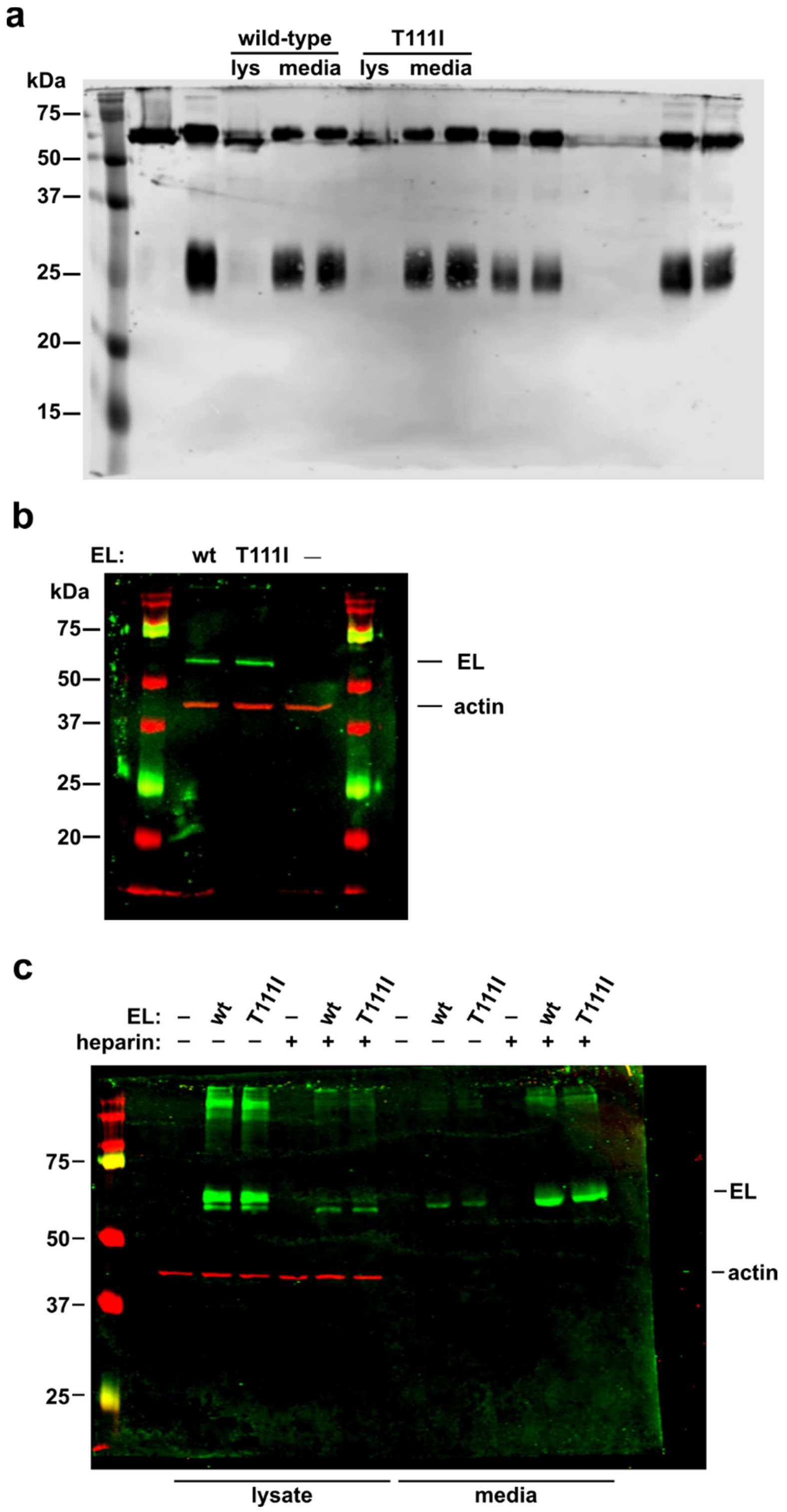

Supplemental Figure 1: Uncropped blots. a) Uncropped version of blot shown in Figure 1b. Expression and secretion of wild-type and T111I EL. Western blot shows expression of EL in the lysate (lys) and media of 293T cells transfected with wild-type or T111I EL. Unlabeled lanes represent samples not used in this study. **b)** Uncropped version of blot shown in Figure 5a. Expression of WT and T111I EL from transduced RHMVEC. Western blot shows expression of EL in the lysate of virally transduced endothelial cells. **c)** Uncropped version of blot shown in Figure 5b.
